# Graphene oxide and its derivatives as promising *In-vitro* bio-imaging platforms

**DOI:** 10.1038/s41598-020-75090-w

**Published:** 2020-10-22

**Authors:** Yasaman Esmaeili, Elham Bidram, Ali Zarrabi, Abbas Amini, Chun Cheng

**Affiliations:** 1 Faculty of Advanced Sciences and Technologies, University of Isfahan, Isfahan, Iran; 2 Biosensor Research Center, Department of Biomaterials, Nanotechnology, and Tissue Engineering, School of Advanced Technologies in Medicine, Isfahan University of Medical Sciences, Isfahan, Iran; 3 Nanotechnology Research and Application Center (SUNUM), Sabanci University, 34956 Tuzla, Istanbul, Turkey; 4 Department of Mechanical Engineering, Australian College of Kuwait, 13015 Mishref, Safat Kuwait; 5 Department of Materials Science and Engineering, Southern University of Science and Technology, Shenzhen, China

**Keywords:** Biochemistry, Chemistry

## Abstract

Intrinsic fluorescence and versatile optical properties of Graphene Oxide (GO) in visible and near-infrared range introduce this nanomaterial as a promising candidate for numerous clinical applications for early-diagnose of diseases. Despite recent progresses in the impact of major features of GO on the photoluminescence properties of GO, their modifications have not yet systematically understood. Here, to study the modification effects on the fluorescence behavior, poly ethylene glycol (PEG) polymer, metal nanoparticles (Au and Fe_3_O_4_) and folic acid (FA) molecules were used to functionalize the GO surface. The fluorescence performances in different environments (water, DMEM cell media and phosphate buffer with two different pH values) were assessed through fluorescence spectroscopy and fluorescent microscopy, while Fourier-transform infrared spectroscopy (FTIR) and X-ray diffraction (XRD) and Scanning electron microscopy (SEM) were utilized to evaluate the modifications of chemical structures. The modification of GO with desired molecules improved the photoluminescence property. The synthesized platforms of GO-PEG, GO-PEG-Au, GO-PEG-Fe_3_O_4_ and GO-PEG-FA illustrated emissions in three main fluorescence regions (blue, green and red), suitable for tracing and bio-imaging purposes. Considering MTT results, these platforms potentially positioned themselves as non-invasive optical sensors for the diagnosis alternatives of traditional imaging agents.

## Introduction

Graphene Oxide (GO) with two-dimensional (2D) network and heterogeneous chemical and electronic structure has attracted considerable attentions in recent years^1,2^. GO possesses numerous favorable properties, such as high mechanical strength, photo-stability, easy surface modification, and excitation-wavelength-dependent photoluminescence (PL)^3–6^. The 2D surface of GO with oxygen-containing functional groups provides a chemically tunable platform to interact with a wide range of biomolecules via covalent/non-covalent interactions, electrostatic forces, π-π absorption and hydrogen bonding^7,8^. Besides, GO consists of sp^2^ and sp^3^ carbons which turns to electronic and optical band gaps for PL phenomenon^9,10^. Each fluorescence peaks in chemically modified GO is a fingerprint of specific electronic transition between the bonding and anti-bonding molecular orbitals^11^. The electronic band structure adjusted by the ratio of sp^2^/sp^3^ orbitals, tunes up the fluorescence property from visible light state to near-infrared (NIR) wavelength range^12,13^. As soon as π electrons are confined in the localized sp^2^ regions (alteration in the localized domains), it further tunes the fluorescence of GO^14^. Here, the distance between charge/energy donor and charge/energy acceptor plays a key role in the electronic energy transitions and fluorescence behavior^15,16^. In fact, small molecular distance between GO and adjacent fluorophores (~ 6 nm) quenches the fluorescence emission of GO when it acts as an energy donor^17^. On the other hand, GO as an energy acceptor quenches the fluorescence of fluorophores when the molecular distance is extended to ~ 30 nm^18^.

The variety of functional groups, lateral size, localized domains and dopants of solvents can effectively influence the electronic energy transitions and fluorescence property of GO^19^. Based on a study by Ming, the position and intensity of fluorescence peak were distorted when GO was treated by KOH or HNO_3_, this respectively resulted in blue-shift and red-shift phenomena, due to the enrichment of GO with OH or COOH groups^11^. Moreover, the PL behavior of GO is typically dependent on the pH values of solvents, causing an excited-state proton transfer^20,21^. Upon the excitation, the protonation of COO^-^ to COOH results in the broad fluorescence peak, while the intensity is relatively quenched. Under basic conditions, however, the fluorescence intensity may be recovered due to the produced COO^-^ moieties^20,21^. Besides, the fluorescence emission and intensity of GO are further affected by the size, chemical functional groups, oxidation degree and other related factors^22,23^. GO can perform as an efficient quencher through either charge transfer or resonance energy^24^. In the customized dopamine biosensor utilizing GO fluorescence, the detection was based on the charge transfer between GO and dopamine quenching the fluorescence property of GO^25,26^. Based on another study, the GO sensor platform was used for the detection of metal cations^27,28^, where GO was applied as a fluorophore (electron donor) and the metal ions acted as the electron acceptor^29,30^.

The nature of fluorescence property of fluorophores, such as quantum dots and organic dyes, basically quenches by GO through the Fluorescence Resonance Energy Transfer (FRET)^31,32^. According to recent findings, quencher GO possesses a large number of bonding sites through oxygen-containing groups, which is ideal for targeting and delivery purposes^33,34^. The captured molecules on the surface of GO, including the fluorophore-labeled single stranded aptamers, double-stranded DNA molecules or antibodies/antigens^35–37^ in the optimized distance, would turn on the fluorescence^38^. The dual role of GO, as a fluorophore and quencher, introduces that as a potential polymer for developing new sensors with multiplex detection capability, however, the broad fluorescence emission restricted its bio-imaging performances^39,40^. Proper modification of GO, using polymers, noble-metal nanoparticles and molecules, improves its fluorescence emission for definite detection/biosensing purposes^41,42^. For targeting delivery purposes, the modification with polymers increases the hydrophilicity and circulation of GO through the biological environment and reduces the steric hindrance between the targeting ligand and biomarker^43,44^. There are some reports on several polymers, including polyethyleneimine-polylactide (PEI-PLA) and polyethylene glycol (PEG), with bright and multi-color auto-fluorescence properties for theranostic systems^45,46^. Metal nanoparticles, on the other hand, are widely used to construct structures with unique electric, catalytic and photonic properties, such as local surface plasmon resonance (LSPR)^47^, surface-enhanced Raman scattering (SERS)^48,49^, and surface-enhanced fluorescence (SEF)^50^. Noble-metal nanoparticles-modified GO produce nano-platform with numerous applications in targeting, delivery, therapy, imaging and sensing properties^51,52^. Among various targeting ligands in delivery conjugates, the fluorescence spectroscopic behavior of folic acid (FA) was seen to be suitable for targeted imaging approaches^53^. These auto-fluorescence molecules had a large absorption cross-section bond overlapping with GO spectrum, where the emission peaks shifted from UV to NIR region, improving the detected fluorescence behavior^54^. These findings triggered our research interests towards investigating derivetized GO as a new theranostic agent for biomedical approaches. The fluorescent biosensors, used in the near-IR region by facilitating the effective interference-free signals, can avoid the interferences (e.g., auto-fluorescence and scattering light) in biological environments^55^.

## Experimental

### Materials

GO, 1-ethyl-3-(3-dimethylaminopropyl) carbodiimide (EDC), N, N′-Dicyclohexylcarbodiimide (DCC), Chloroauric acid (HAuCl_4_), and Folic acid (FA), 3-(4,5-Dimethylthiazol-2-yl)-2,5-diphenyltetrazolium bromide (MTT) and fetal bovine serum (FBS) were purchased from Sigma Aldrich, USA. Polyethylene glycol (PEG_6000_) was purchased from ROTH, Germany. 4- Dimethylaminopyridine (DMAP), Dimethyl sulfoxide (DMSO), sodium citrate, FeCl_3_·6H2O and FeCl_2_·6H2O received from Merck, Germany. Dulbecco’s Modified Eagle Medium (DMEM), Penicillin/Streptomycin (Pen/Strep), Trypsin–EDTA enzyme were provided by Gibco, USA.

### Methods

#### Synthesis of nano-conjugates

##### GO-PEG

To prepare PEGylated graphene oxide (GO-PEG), GO was acylated using EDC and DMAP connecting PEG molecules via ester bonds^56–59^. In brief, 50 mg GO was dispersed in 100 ml deionized water (DI) with 50 mg EDC and DMAP under bath sonication followed by adding 100 mg PEG6000 at room temperature. The solution was kept stirring vigorously at 60 °C overnight. The final product, GO-PEG, was washed and purified in a dialysis tube (MW cut off: 12,000 KDa).

##### GO-PEG-Fe_3_O_4_

GO-PEG (400 mg) was added to 70 mL of 0.1 M NaOH solution and sonicated at room temperature for 45 min. The desired product was separated by centrifuging and washed with DI water for several times to adjust the pH to 6. The volume of 20 ml of degassed water was added to the isolated product and dispersed by an ultrasonic bath for 30 min. A solution of FeCl_3_·6H_2_O (48 mg) and FeCl_2_·4H_2_O (17.6 mg) in 5 ml degassed water was mixed dropwise with the suspension at 60 °C in an ultrasonic bath for 60 min. Then, ammonium hydroxide solution (23%) was added to the mixture through a funnel to adjust pH to 11–12. The final product, GO-PEG-Fe_3_O_4_, was separated by a magnet and washed several times^60^.

##### GO-PEG-Au

AuNPs were synthesized separately using Turkevich method based on reduction of the HAuCl_4_ by citrate in water^61^. In brief, chloroauric acid solution (HAuCl_4_) (200 ml of 0.01 wt. %) was heated for 20 min and refluxed in a 500 ml-round-bottom flask using a temperature-controlled hot plate under continuous stirring. A 4.5-ml aliquot of 1 wt. % sodium citrate solution was heated for 20 min and added to the boiling chloroauric acid solution, while heating under reflux for 15 min to reach the complete reaction. Then, the solution was allowed to cool down to room temperature with continuous stirring to yield citrate-capped AuNPs. In the following step, the synthesized gold nanoparticles were added to GO-PEG solution under sonication for 2 h; another stirring process was performed subsequently for further 5 h at room temperature. The final product, GO-PEG-Au, was obtained after the crude product was purified through dialysis (MW cut off 12,000 KDa).

##### GO-PEG-FA

GO-PEG (400 mg) was dispersed in 250 ml DMSO in a bath sonication followed by adding DCC (45 mg), DMAP (25 mg) and FA (67 mg) at room temperature. The solution was kept stirring vigorously at 60 °C under nitrogen gas for 36 h. The final product, GO-PEG-FA, was washed and purified in a dialysis tube (MW cut off 12,000 KDa)^62^.

#### Characterization nano-conjugates

The chemical states and physical properties of nano-conjugates were studied using Fourier-transform infrared spectroscopy (FTIR) (JACSO 6300, Japan) and X-ray diffraction (XRD) (Asenware AW-DX300, England). Scanning electron microscopy (SEM, TESCAN MIR3, Czech Republic) was used to study the morphology of synthesized conjugations.

#### Fluorescence study

The fluorescence intensity of each conjugation was assessed through three different solvents (water, cell media, phosphate buffer at two pH values of 6.6 and 7.4) using a Luminescence Spectrometer (LS 55). The multicolor fluorescence property of conjugations was further assessed by a fluorescence microscopy.

#### Biocompatibility study

Cell viability was measured using MTT assay. Briefly, MCF-7 as a tumor cell line and L-929 as a non-tumor cell line were cultured in DMEM media with 10% (v/v) FBS and 1% pen/strep. The cells were incubated at 37 °C in a humidified incubator containing 4% CO_2_ in 25 cm^3^ tissue culture-treated flasks. MCF-7 and L-929 (10^4^ cells/well) were seeded at 96-well plates in full medium and incubated overnight at 37 °C. Two cell lines were treated with GO-PEG, GO-PEG-Fe_3_O_4_, GO-PEG-AuNPs, GO-PEG-FA at different concentrations (200, 100, 50 μg.ml^-1^), as well as the untreated cells as the negative control. After 48 h, the supernatant was removed and cells were washed immediately by PBS. An aliquot of 90 μL DMEM and 10 μL MTT stock solution were subsequently added to each well incubated for 4 h at 37 °C. In the following stage, the MTT solution was removed, while 100 μL DMSO added to each well incubated for further 40 min at 37 °C. Finally, the absorbance was read at 490 nm with an ELISA reader (Biorad-USA). The experiment was performed in triplicate.

#### Statistical analysis

SPSS software (version 21, parametric analysis of variance [ANOVA (Tukey)]) was used for quantitative data analysis and results are reported as mean values ± standard deviation (SD) with significant value at *p* ≤ 0.05.

## Results and discussion

### Characterization

#### GO-PEG

**Figure 1:**
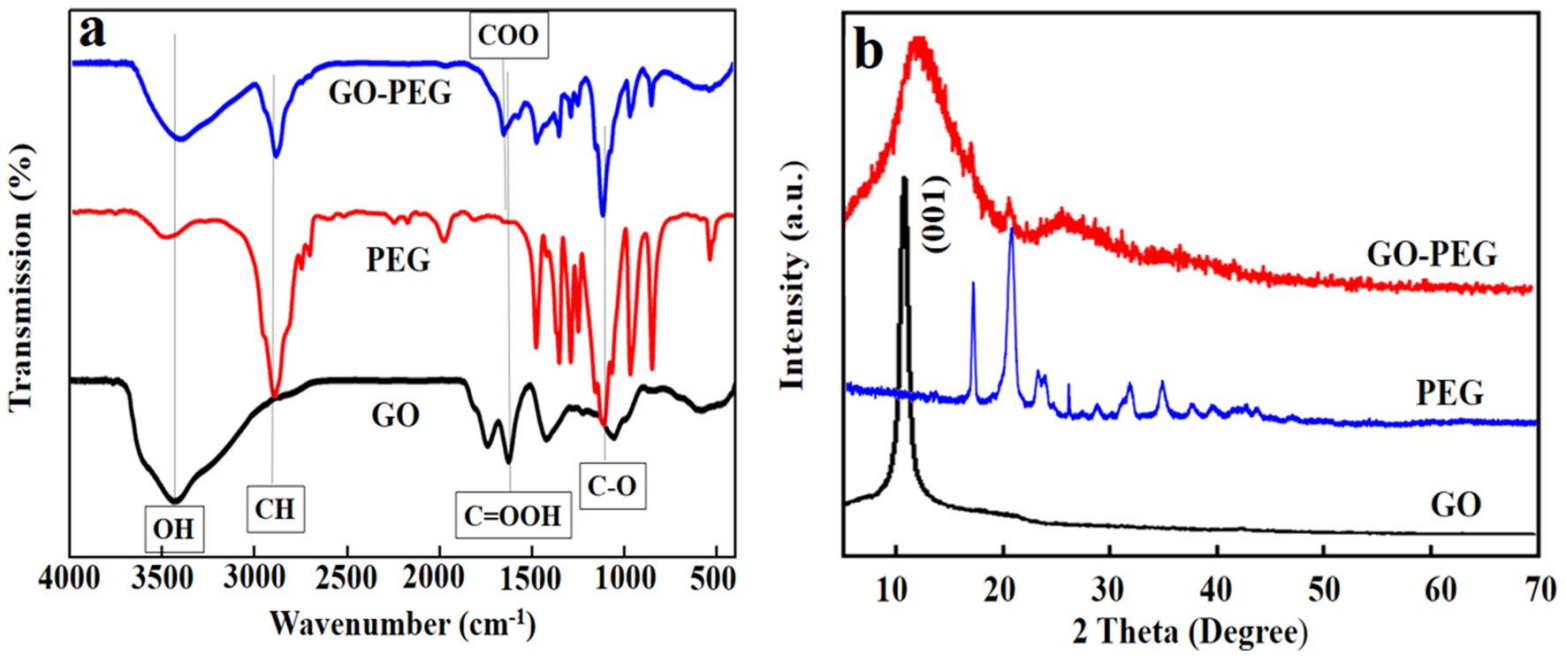
**(a)** FTIR of GO, PEG and GO-PEG, (**b)** XRD patterns of GO, PEG and GO-PEG.

The chemical composition and interfacial interaction of the GO-PEG nano-conjugate were characterized by FTIR. Figure 1a represents the FTIR spectra of GO, PEG and GO-PEG where the broad peaks of GO at ~ 3000 to 3700 cm^-1^ are related to the stretching vibrations of hydroxyl (O–H) group. The peak at ~ 1737 cm^-1^ is attributed to the stretching vibrations of C = O band of carbonyl groups demonstrating the lack of carboxyl groups on the surface of GO^63^ . The PEG related bands are attributed to the stretching vibrations of C–H , C = O, and bending vibration of C–O, respectively, at 2847, 1647, and 1104 cm^-1^. The deformation vibration of C–H bonds are determined at 1468 and 1342 cm^-1^, the bending vibration of O–H at 1280 and 1242 cm^-1^, and C–O stretching vibration at 1149 cm^-1^^64^. Considering the FTIR spectrum of GO-PEG, the major absorption peaks of PEG and GO are preserved in GO-PEG with a trivial shift of peak positions and relative change of intensity. Additionally, as there are C = O and O–H groups in both PEG and GO, FT-IR curve represents the ester bonding between PEG carboxyl groups and GO. These are the signatures of functional groups of GO, PEG and GO-PEG which confirm the successful conjugation of GO-PEG.

The XRD patterns of GO, PEG and GO-PEG are displayed in Fig. 1b. GO has an influence on the arrangement of molecular chain of PEG in the crystal lattice, disturbing the order of its crystallization. This decreases the crystallinity of PEG and concludes in an effective conjugation of PEG to GO nano-sheets by ester bonding.

**Figure 2:**
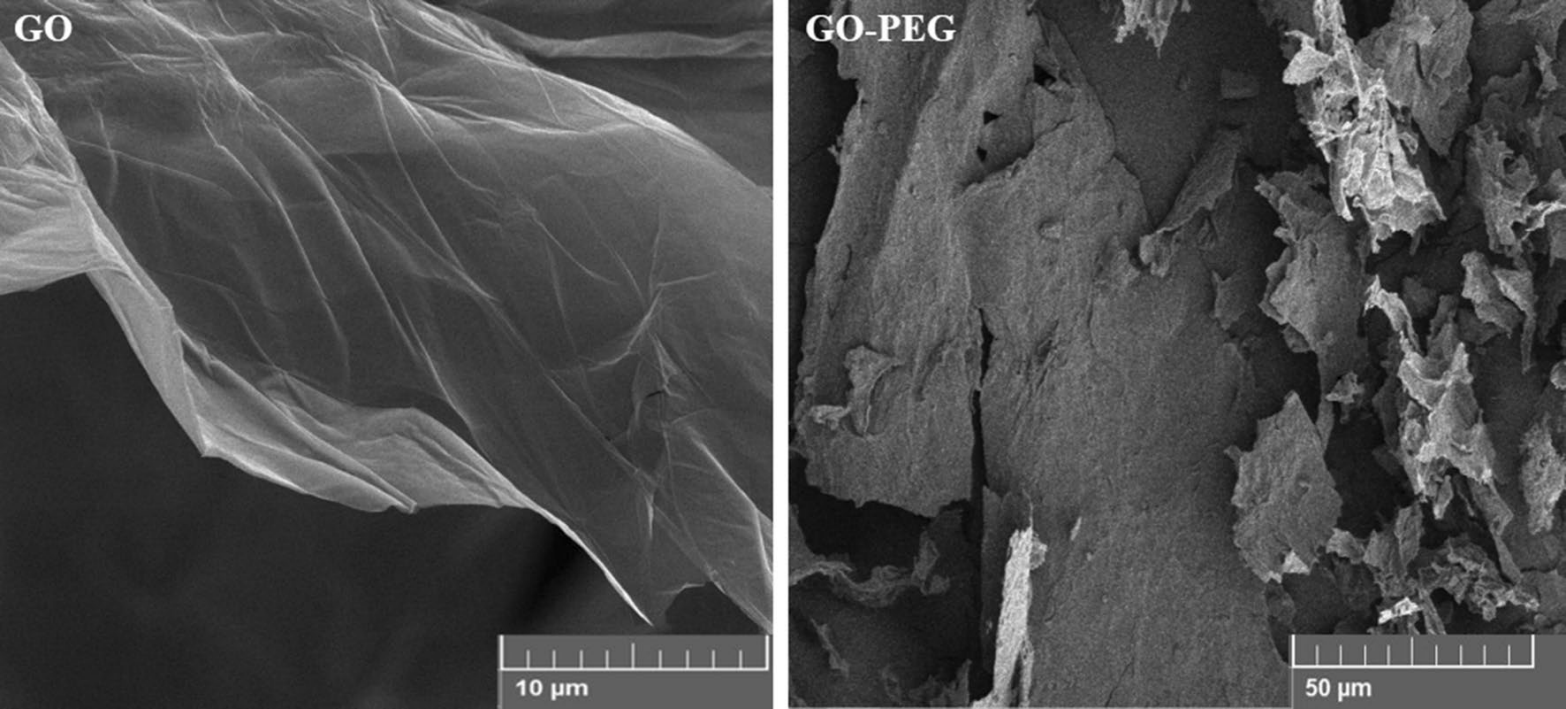
FE-SEM images of GO and GO-PEG.

The XRD pattern of pure GO was analyzed based on a sharp diffraction peak at 2θ = 10.4°, corresponded to the (001) crystalline plane diffraction peak of GO^65^. The XRD diffraction pattern of PEG was confirmed by the characteristic diffraction peaks at 19.2°, 23.3° and 26.4°^66^. The XRD diffraction pattern of GO-PEG represented the amorphic pattern of the nano-conjugate at 2θ = 15°. The diffraction peak at 2θ = 29° was expanded, indicating more amorphous structure of nano-conjugate. The morphology of GO and GO-PEG was further assessed using FE-SEM; GO had a layered distribution stacking together in a flocculent manner. The surface was relatively flat while the GO-PEG curled to an irregular shape due to the inter-molecular ester bonding (Fig. 2).

#### GO-PEG-Fe_3_O_4_

**Figure 3:**
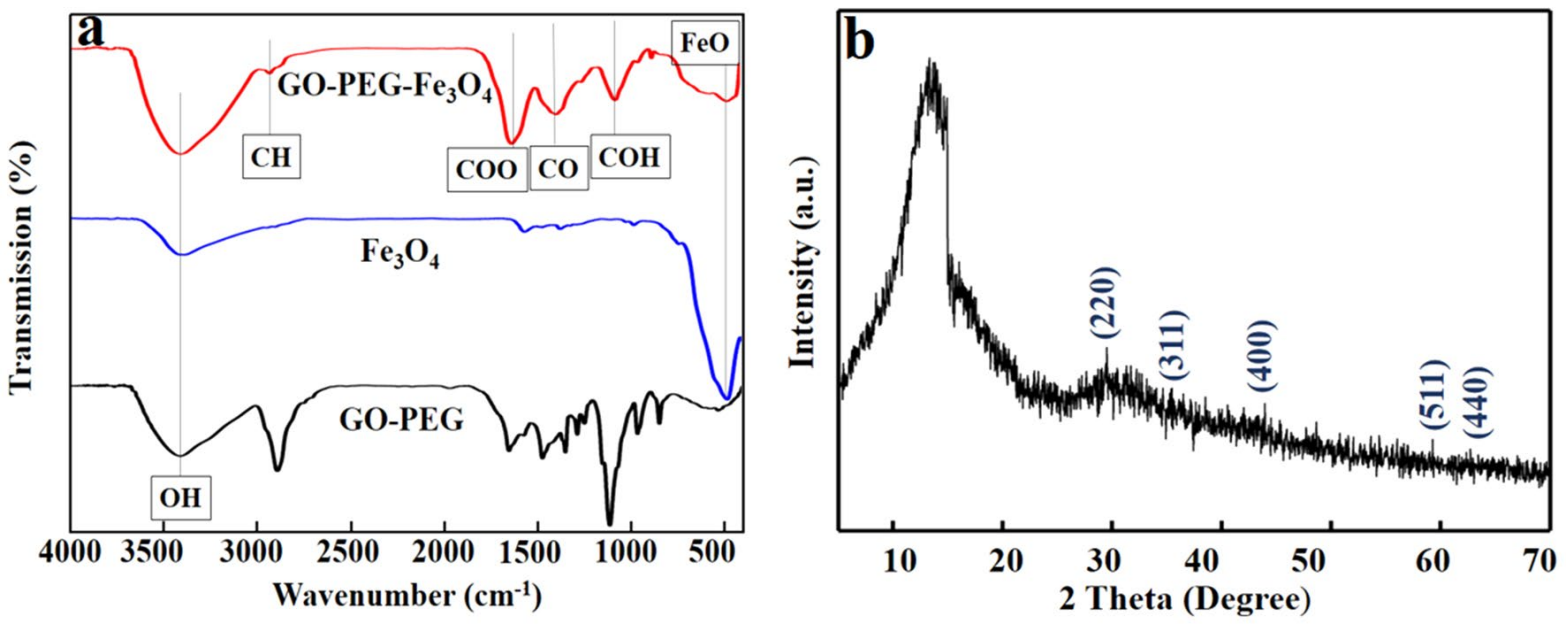
**(a)** FTIR pattern of GO-PEG-Fe_3_O_4_, (**b)** XRD patterns of GO-PEG-Fe_3_O_4_.

In Fig. 3a, the sharp peak in FTIR pattern of GO-PEG-Fe_3_O_4_ at 577 cm^−1^ is attributed to Fe–O bond of pure Fe_3_O_4_ nanoparticles^67^. Broad bonds at ~ 1383, 1065, 1623 and 3300 cm^−1^ are corresponded to C–O–C, C–OH, C = O and O–H groups, respectively^68^. The Fe–O bond at 577 cm^-1^ provides strong evidence that the surface of GO-PEG was functionalized with Fe_3_O_4_. The crystallographic structure of GO-PEG-Fe_3_O_4_ was assessed by the XRD pattern in Fig. 3b. The XRD peaks corresponding to Fe_3_O_4_, marked with the indices of (220), (311), (400), (511) and (440), are similar to those reported for Fe_3_O_4_ nanoparticles in^68^; These results are the other pieces of evidence for the successful grafting of Fe_3_O_4_ on the surface of GO-PEG.

**Figure 4:**
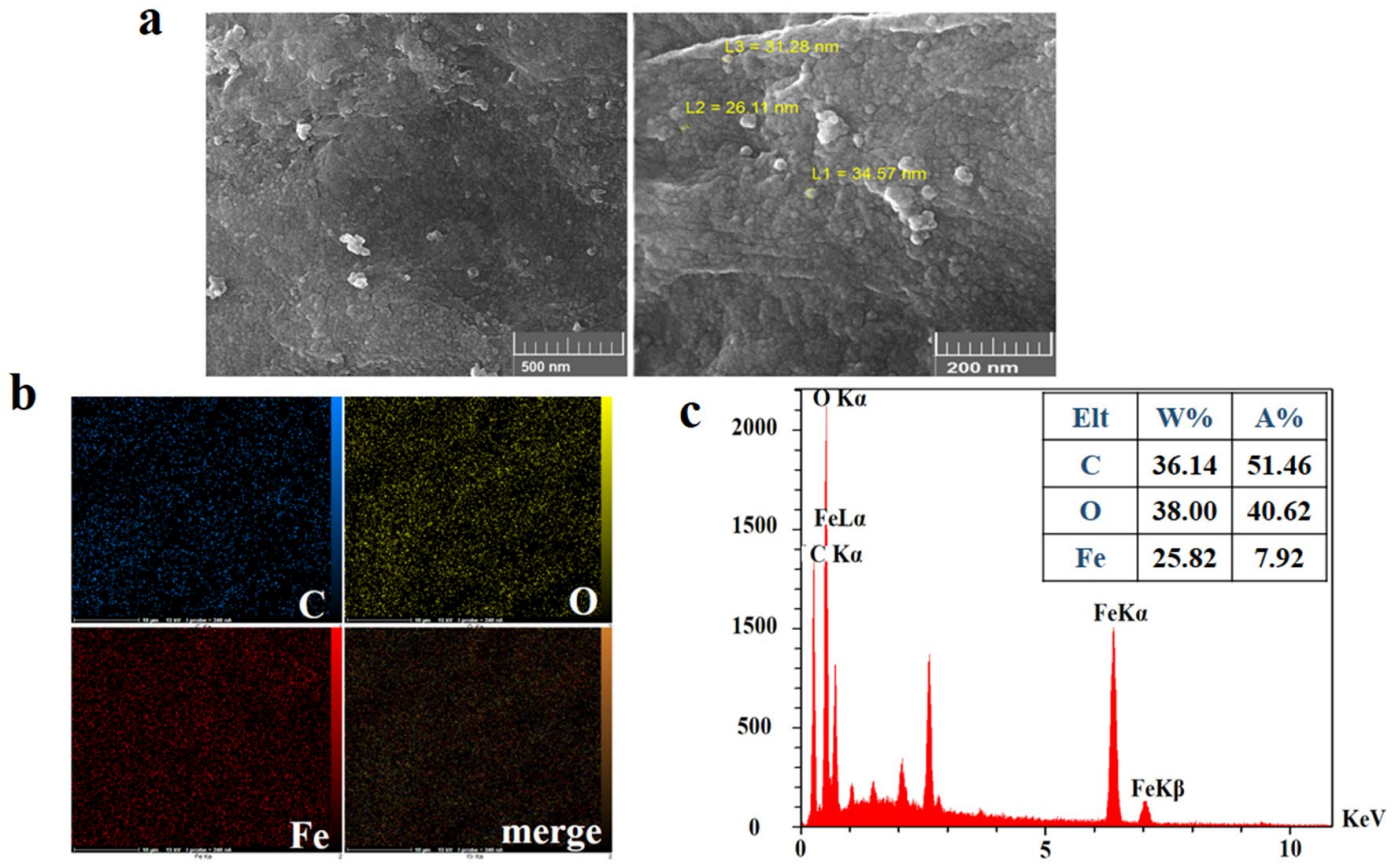
**(a)** FE-SEM images of GO-PEG-Fe_3_O_4_ conjugates, Fe_3_O_4_ distribution with an average size of 27–36 nm on the surface of GO-PEG (**b)** EDX mapping, and (**c)** EDS analysis indicating the element distribution on the surface of GO-PEG- Fe_3_O_4_.

Figure 4a shows the FE-SEM micrographs of GO-PEG-Fe_3_O_4_ where the dispersed Fe_3_O_4_ NPs on the surface of GO-PEG has an average size of ~ 26–34 nm. The meaningful picture of element distribution on the surface verified the presence of carbon, oxygen and iron elements throughout the surface of GO-PEG- Fe_3_O_4_ (Fig. 4b). EDX spectra further showed the corresponding peaks of carbon (C) oxygen (O) and iron (Fe) in the final conjugate (Fig. 4c).

#### GO-PEG-Au

**Figure 5:**
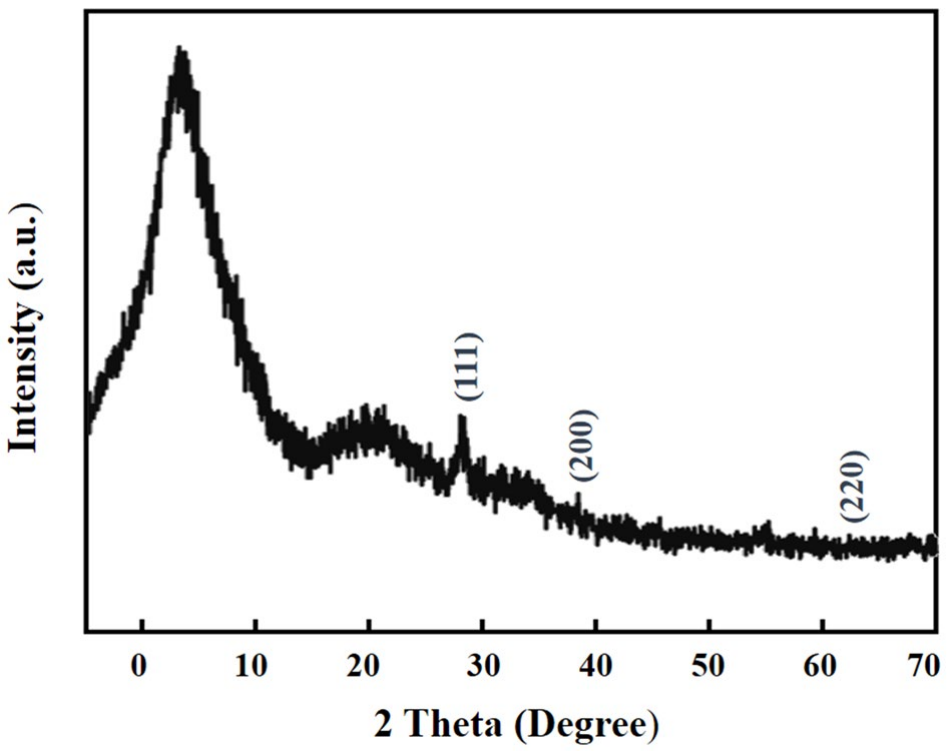
XRD patterns of GO-PEG-Au conjugate.

The crystallographic structure of synthesized GO-PEG-Au was studied using XRD (Fig. 5) with distinguished peaks at 38.2°, 44.59° and 64.7° corresponded to (220), (200) and (111) planes, respectively, confirming the formation of AuNPs in the nano-conjugate^69^.

**Figure 6:**
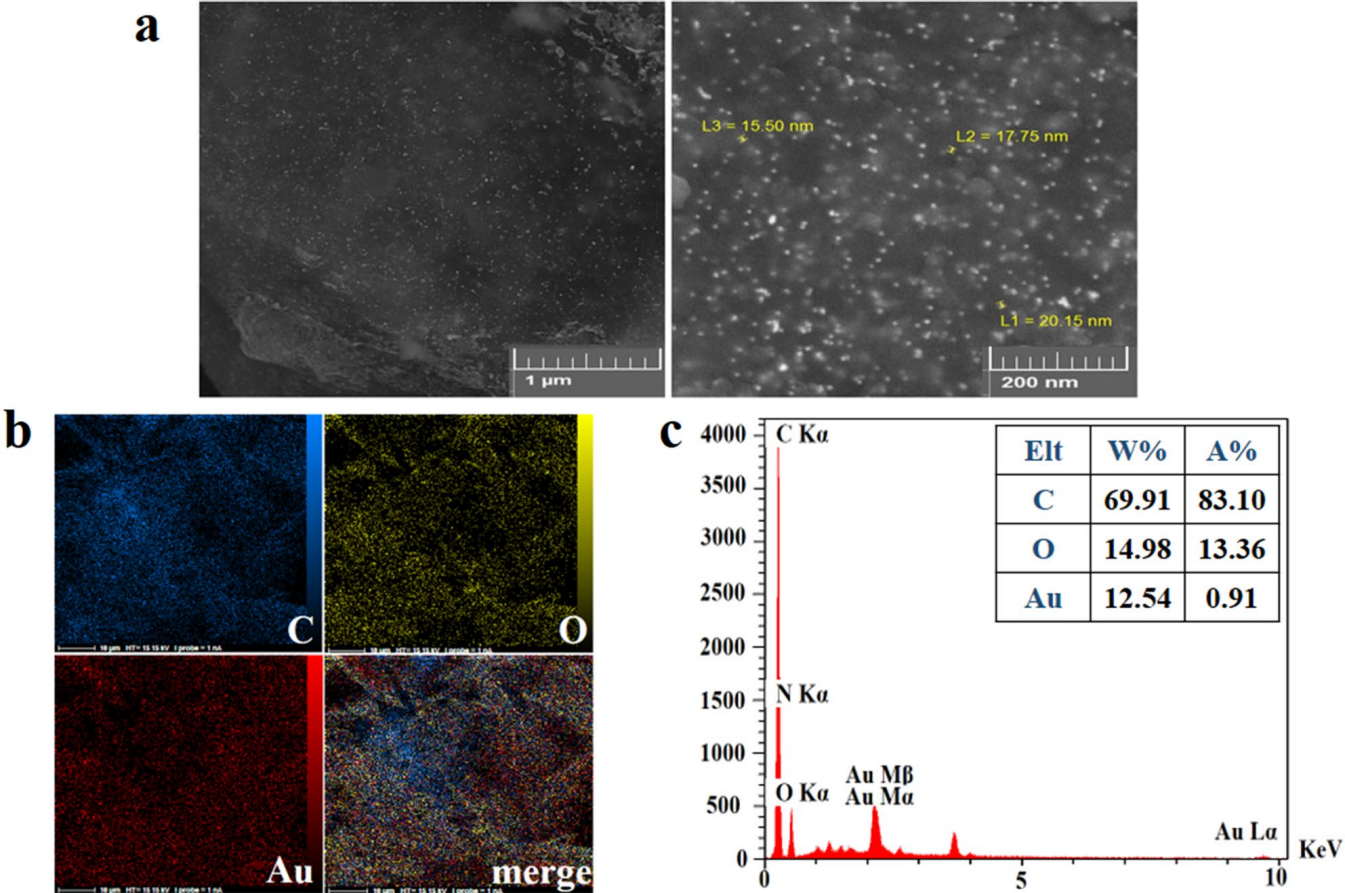
**(a)** FE-SEM images of GO-PEG-Au conjugates showing AuNPs distribution with an average size of 15–17 nm on the surface of GO-PEG, (**b)** EDX mapping, and (**c)** EDS analysis indicating the element distribution on surface of GO-PEG-Au.

Figure 6a shows the FE-SEM micrographs of GO-PEG-Au with the uniform dispersion of AuNPs on the surface of GO-PEG and an average size of ~ 15–20 nm. The chemical composition of GO-PEG-Au was characterized through EDX analysis. The meaningful picture of element distribution on the surface verified the presence of carbon, oxygen and gold elements throughout the surface of GO-PEG-Au (Fig. 6b). EDX spectra further showed the corresponding peaks of C, O and gold (Au) in the final conjugate (Fig. 6c).

#### GO-PEG-FA

**Figure 7:**
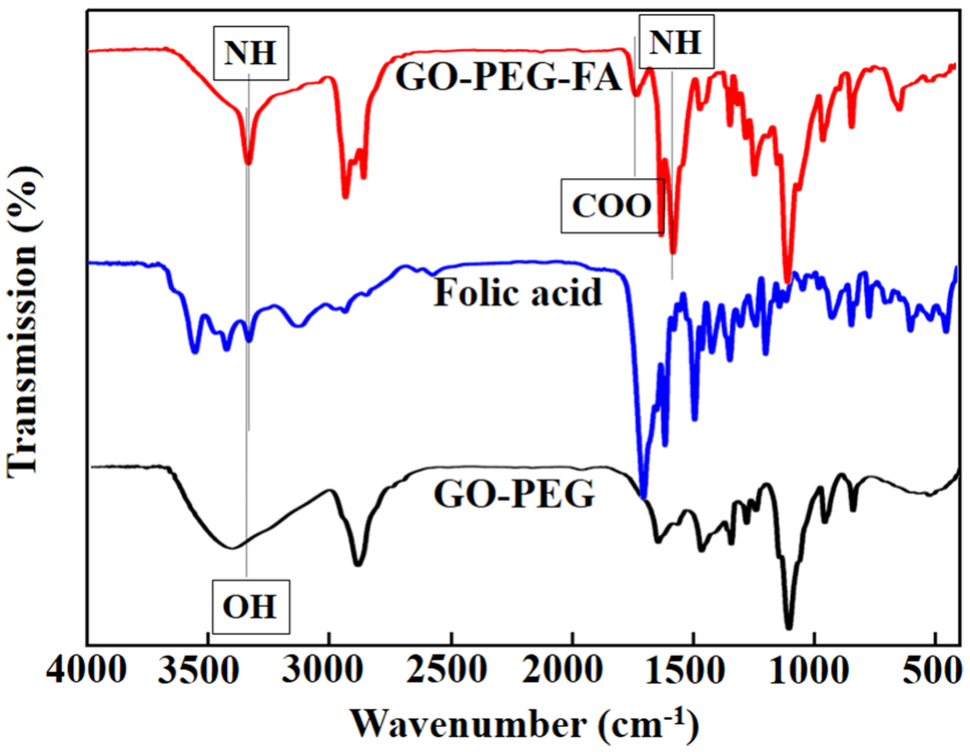
FTIR pattern of GO-PEG-FA conjugate shows the covalent interaction between FA and GO-PEG.

The grafting GO-PEG with FA led to the introduction of new absorbance peaks in the FTIR pattern of GO-PEG-FA at 1640 cm^−1^ and 3400–3500 cm^−1^, representing the N–H group, and the peak at 1400 cm^−1^ corresponded to the aromatic ring stretch of the pteridine ring and ρ-amino benzoic acid moieties of FA^70^. Another peak at ~ 1700 cm^−1^ was an evidence of the ester bonding between FA and GO-PEG surface (Fig. 7).

**Figure 8:**
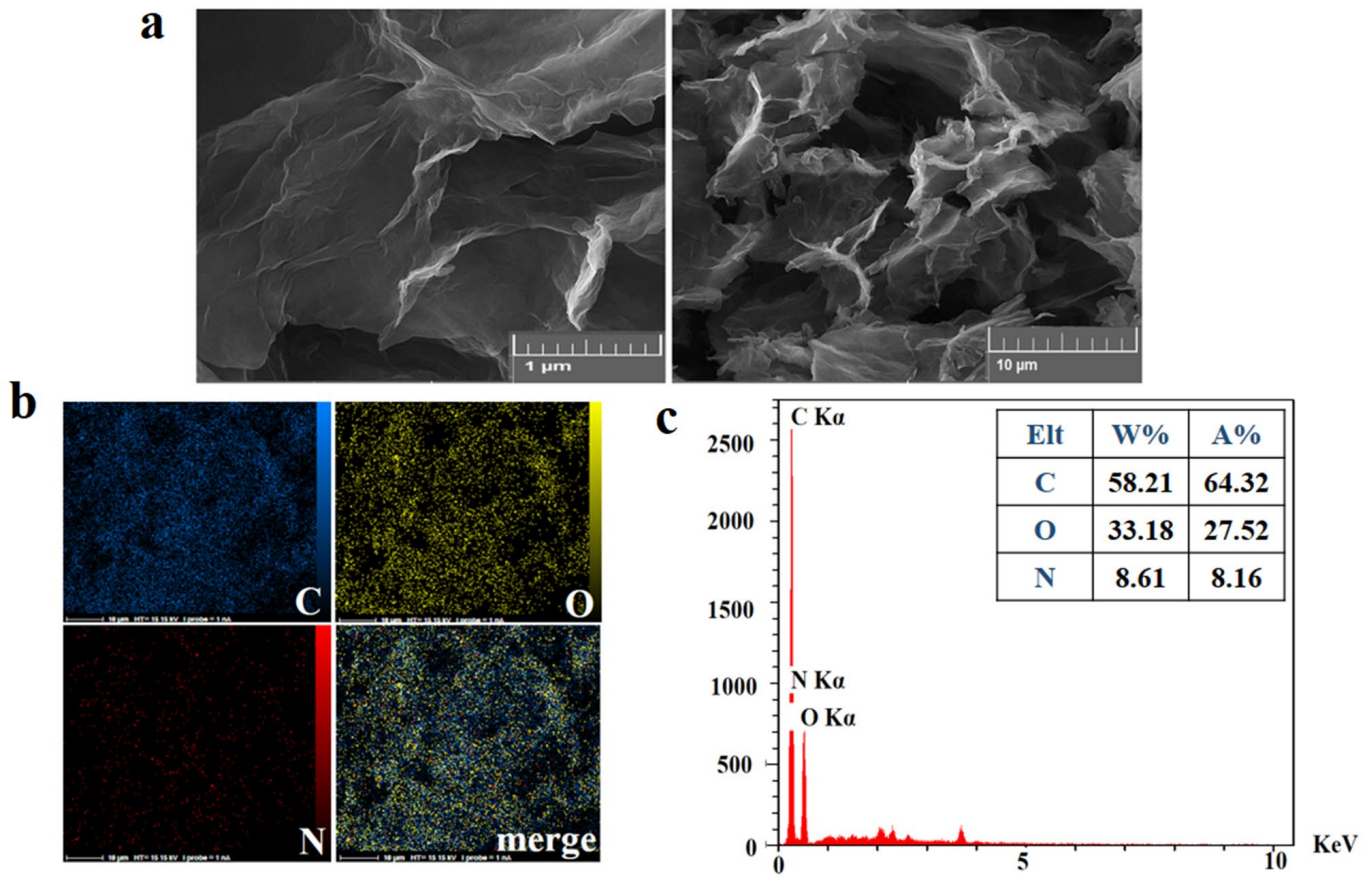
**(a)** FE-SEM images of GO-PEG-FA conjugate, (**b)** EDX mapping, and (**c)** EDS analysis illustrates the elemental distribution on the surface of GO-PEG-FA conjugate.

FE-SEM images of the modified GO revealed a sheet like morphology with wrinkled structure for FA-functionalized GO-PEG (Fig. 8a)^71^. EDX analysis presented a meaningful picture for the element distribution on the surface of conjugate, verifying the presence of nitrogen in the folic acid structure (Fig. 8b). EDX spectra contained the related peaks of C, O and nitrogen (N) (Fig. 8c).

### Fluorescence study

Considering the special conditions of biomedical applications and tumors environment, pH level plays a critical role in biomedical approaches. To study the solution effect on the intensity and pattern of detected fluorescence, GO derivatives were dissolved in three different solvents (water, PBS and DMEM cell media). Acidic pH particularly needs to be considered for the system design for imaging or tracking purposes, either inside (endosomes) or outside (tumor area) the cells. As discussed in the following sessions, apart from the type of functionalization, the solvent considerably affects the emitted fluorescence^72^.

#### Emission spectra at the excitation wavelength of 300 nm

**Figure 9:**
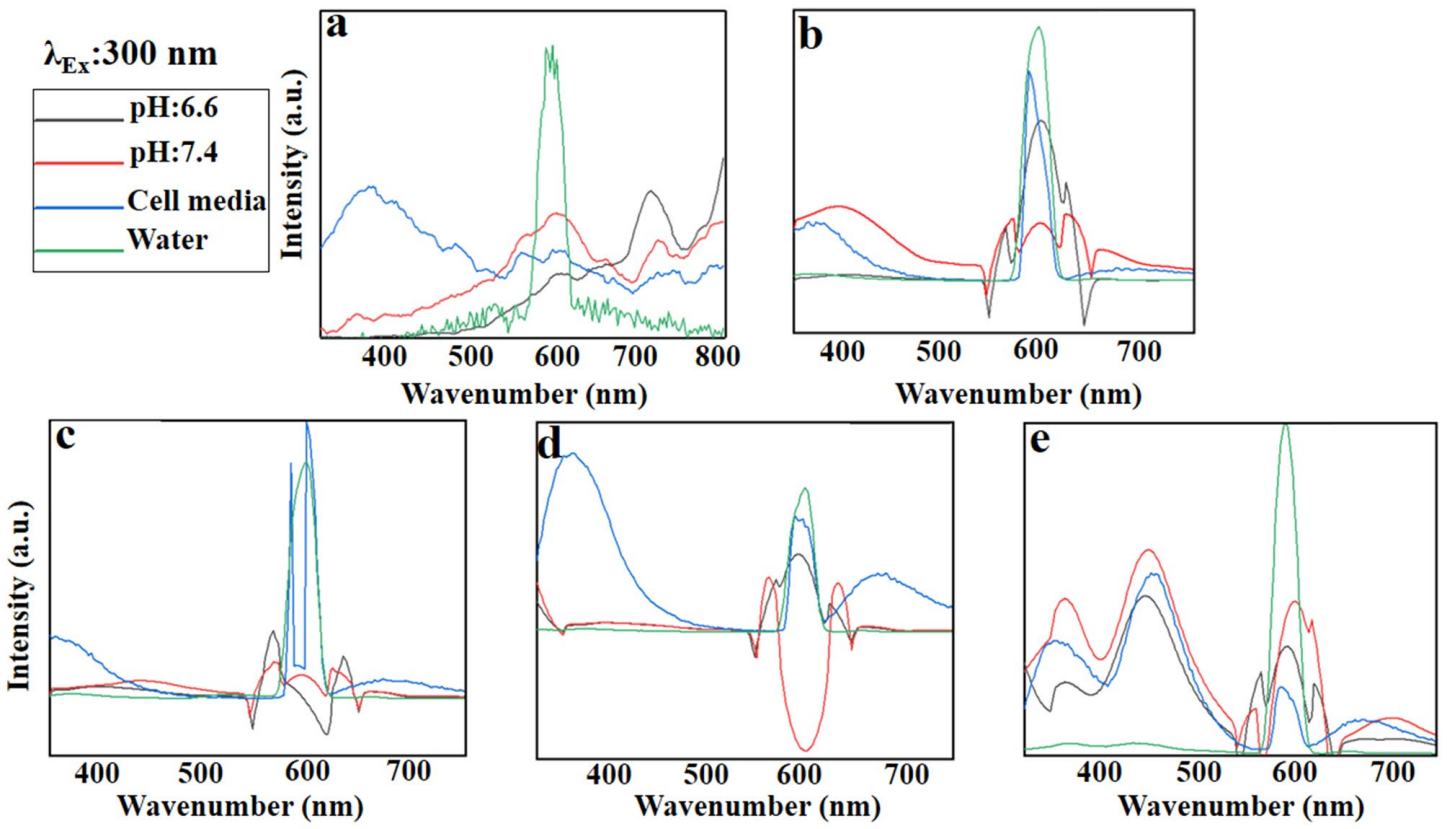
Emission patterns of (**a)** GO, (**b)** GO-PEG, (**c)** GO-PEG-Fe_3_O_4_, (**d)** GO-PEG-Au, and (**e)** GO-PEG-FA at the excitation wavelength of 300 nm in water, cell media (DMEM) and PBS, at two different pH values (pH 6.6 and 7.4).

Pure GO in water indicates the maximum emission at ~ 595 nm with two broad emission peaks at 400–550 and 650–800 nm. In the DMEM cell media, the maximum emission occurs at ~ 400 nm, while by the variation in pH values it shifts to the red region; it is maximized at 610 nm in natural pH (PBS) and at 730 nm in acidic media (Fig. 9a). GO becomes the charge donor when modified with PEG (GO-PEG), its broad fluorescence spectra are quenched in water at the range of 450–550 and 650–750 nm. In return, GO acts as the energy acceptor quenching the emission peaks of PEG (energy donor) at 508 and 605 nm (Fig. 9b). Likewise, the behavior of PEGylated GO in DMEM cell media is similar to the detected emission in water, while the new peaks at ~ 300–450 and 650–800 nm are attributed to the cell media absorption. At the natural pH (7.4), the fluorescence emissions of both components (GO and PEG) are preserved and the emission peak at 400 nm is blue-shifted, representing GO-PEG as a blue fluorophore. Thus, GO-PEG can be introduced as a turn on/off biosensor in tumor studies, and a tracer for in vitro and in situ imaging.

GO-PEG-Fe_3_O_4_ acts similar to GO-PEG in both water and acidic pH, in which the fluorescence emission curve of GO is quenched in the acidic condition (Fig. 9c). Thereby, it could be applicable as a suitable switch-off biosensor in a tumor area. In cell media, however, the fluorescence of PEG and Fe_3_O_4_ components are both preserved, while the fluorescence of GO is quenched. This fact shows that GO can behave as an energy acceptor and simultaneous energy donor in the cell media, introducing GO-PEG-Fe_3_O_4_ conjugate as a potential tracer for *in-vitro* and in situ tumor imaging. Nevertheless, at natural pH (7.4), the fluorescence would be nearly quenched.

Au accelerates the blue emission (400 nm) with the new peak at red region (700 nm). This introduces GO-Au as a potential candidate for imaging and thermal therapy (Fig. 9d). GO-PEG-Au has a similar behavior in both water and DMEM cell media. As previously mentioned, the new detected peaks at 400 and 700 nm are contributed to the absorption of DMEM cell media. On the contrary, the fluorescence emission of GO would be quenched by Au NPs at both natural and acidic pH values, when GO acts as a charge donor (switch-off biosensor). Figure 9e indicates the interesting patterns of GO-PEG-FA in all various solvents (water, cell media, natural and acidic). Although the blue and red emissions are still detectable with the lower densities, green fluorescence (430–550 nm) is observed with strong peak at 460 nm.

#### Emission spectra at the excitation wavelength of 350 nm

**Figure 10:**
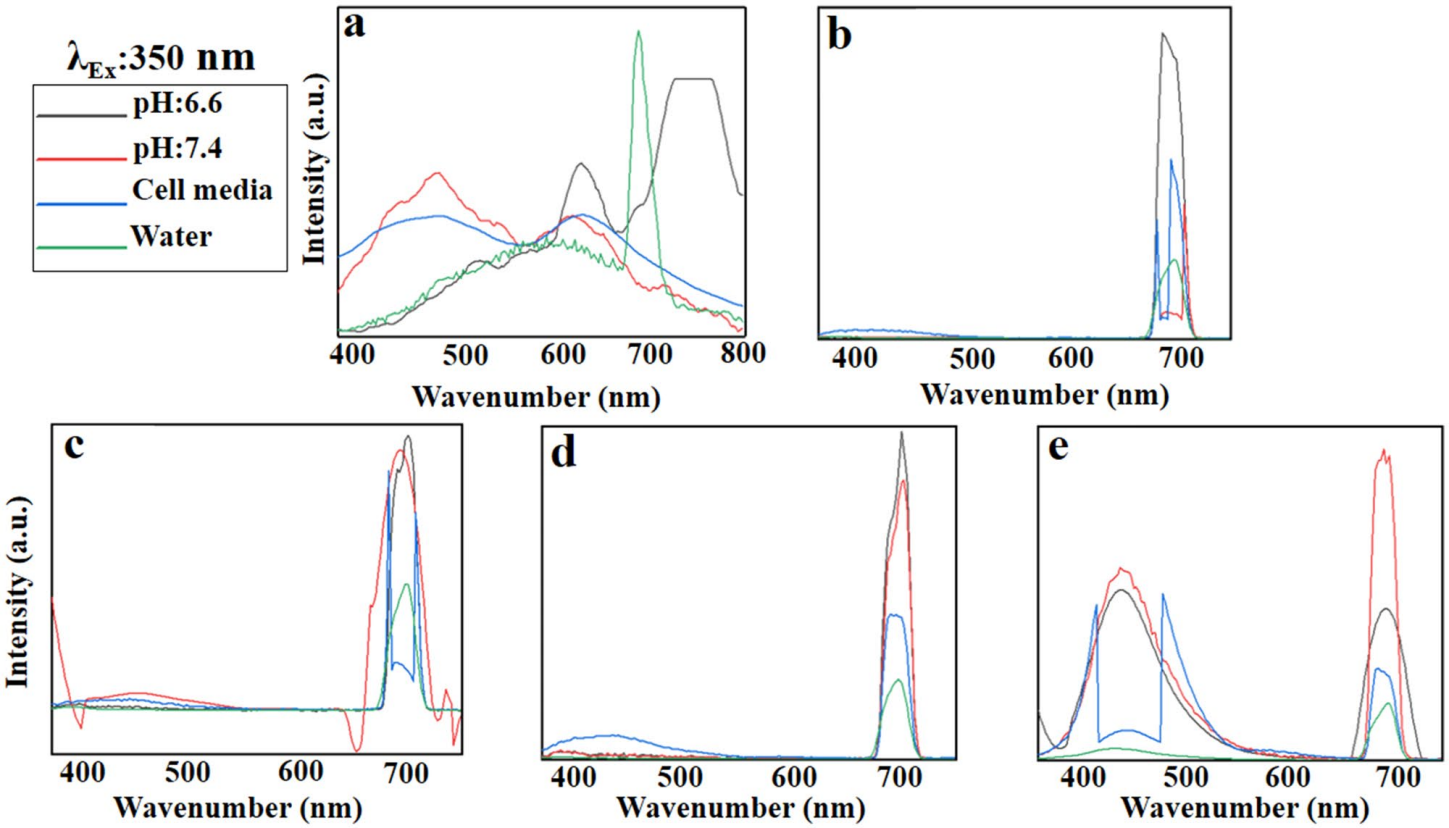
Emission patterns of (**a)** GO, (**b)** GO-PEG, (**c)** GO-PEG-Fe_3_O_4_, (**d)** GO-PEG-Au, and (**e)** GO-PEG-FA at the excitation wavelength of 350 nm in water, cell media (DMEM), and PBS at two different pH values (pH 6.6 and 7.4).

The dissolved GO in water indicates a broad emission at 400–650 nm as well as a sharp peak at 700 nm (Fig. 10a). DMEM cell media and neutral pH affect the trend resulting in a blue shift to 400–550 nm, while acidic pH causes a red-shift with a maximum emission peak at 750 nm. According to Fig. 10b, PEG acts as an energy acceptor quenches the predicted fluorescence of GO. Likewise, the metal nanoparticles have a similar behavior in the wavelength of 350 nm (Fig. 10c, d). Interestingly, FA accelerates the blue emission at 400–500 nm representing a strong fluoresce at the green zone (Fig. 10e), the range appropriated for bio-imaging applications^62^.

#### Emission spectra at excitation wavelength of 430 nm

**Figure 11:**
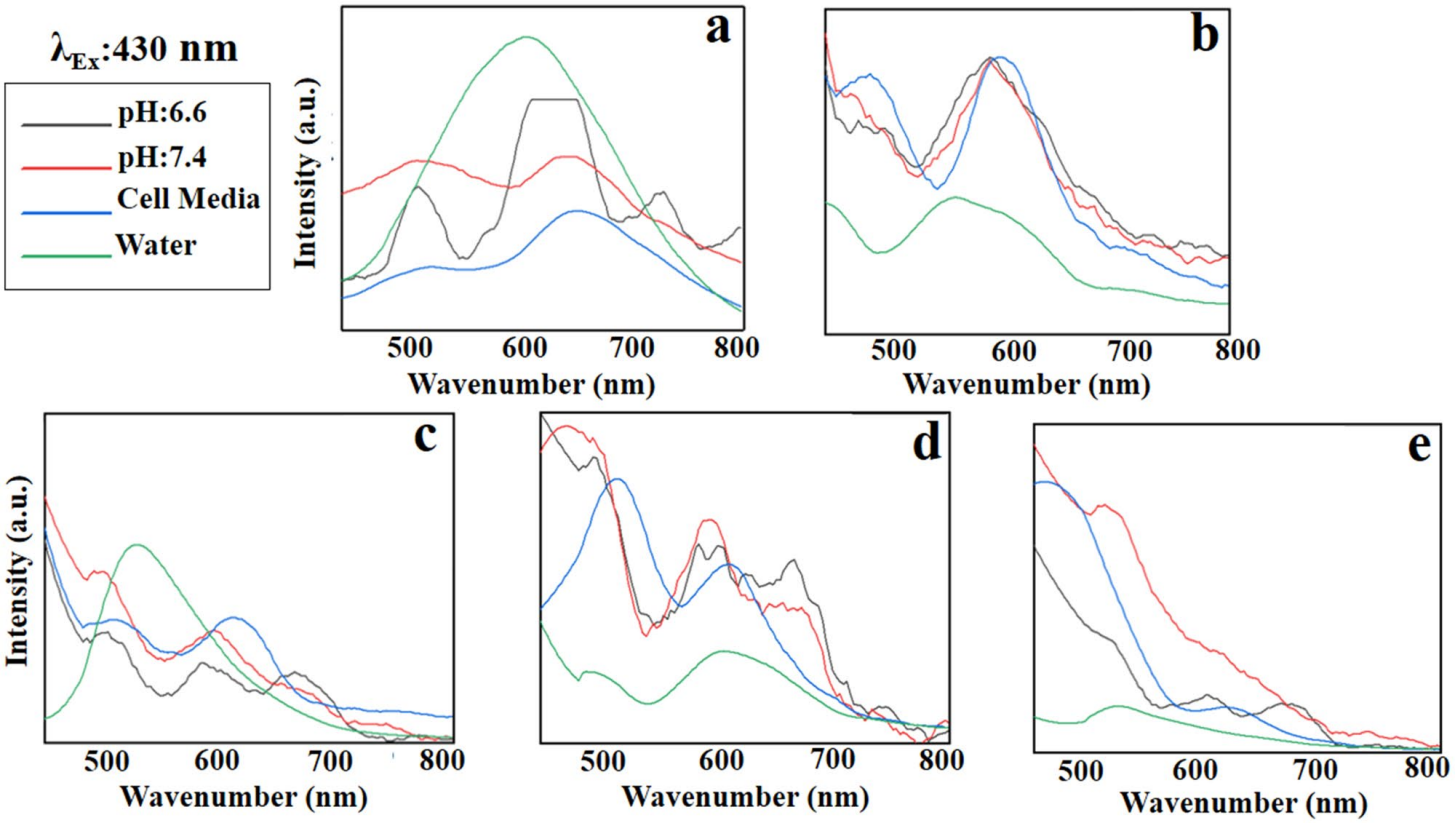
Emission patterns of (**a)** GO, (**b)** GO-PEG, **c)** GO-PEG-Fe_3_O_4_, (**d)** GO-PEG-Au, and (**e)** GO-PEG-FA at the excitation wavelength of 430 nm in water, cell media (DMEM), and PBS at two different pH values (pH 6.6 and 7.4).

At the excitation wavelength of 430 nm, GO has a broad emission (Fig. 11a) that is improved and reinforced with the PEG modification (Fig. 11b) at the red region (560–800 nm) in all solvents. GO-PEG-Fe_3_O_4_ shows a single peak at 547 nm in water, while multiple emission peaks appear at 500, 570 and 650 nm when dissolved in DMEM cell media and PBS (Fig. 11c). Likewise, GO-PEG-Au illustrates the same behavior as GO-PEG-Fe_3_O_4_; while the fluorescence intensity is lower when dissolved in water (Fig. 11d). In the excitation wavelength of 430 nm, GO-PEG-FA does not show any identified emission suitable for imaging purposes (Fig. 11e).

#### Emission spectra at the excitation wavelength of 480 nm

**Figure 12:**
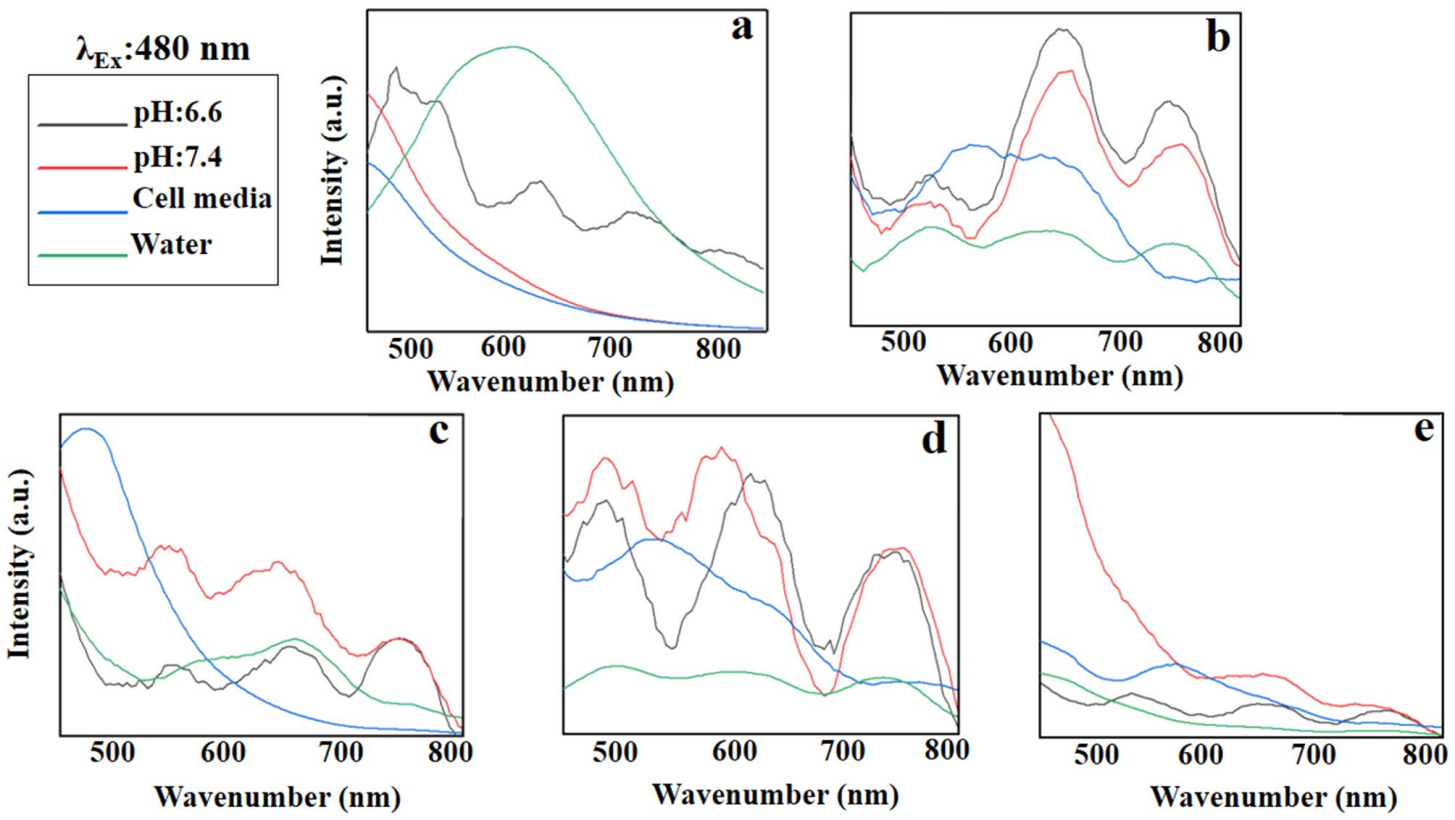
Emission pattern of (**a)** GO, (**b)** GO-PEG, (**c)** GO-PEG-Fe_3_O_4_, (**d)** GO-PEG-Au, and (**e)** GO-PEG-FA at the excitation wavelength of 480 nm in water, cell media (DMEM), PBS at two different pH values (pH 6.6 and 7.4).

GO at the excitation wavelength of 480 nm has an inappropriate fluorescence behavior (Fig. 12a). Functionalization with PEG improves this fluorescence emission and results in a red shift maximized at 650 and 730 nm in natural and acidic conditions, respectively (Fig. 12b). Fe_3_O_4_, however, decreases the fluorescence intensity of the conjugate in acidic condition creating a blue shift in DMEM cell media (Fig. 12c). Due to the broad emission pattern of GO-PEG-Fe_3_O_4_ in water, so, it is not suggested for the detection purposes. Au, on the other hand, increases the fluorescence intensity of the conjugate in both natural and acidic conditions (Fig. 12d).

GO-PEG-Au indicates the multiple emission peaks with low intensity when dissolved in water. GO-PEG-FA decreases the fluorescence intensity of conjugate in all solvents, representing FA as an energy acceptor in the excitation wavelength of 480 nm (Fig. 12e).

**Figure 13:**
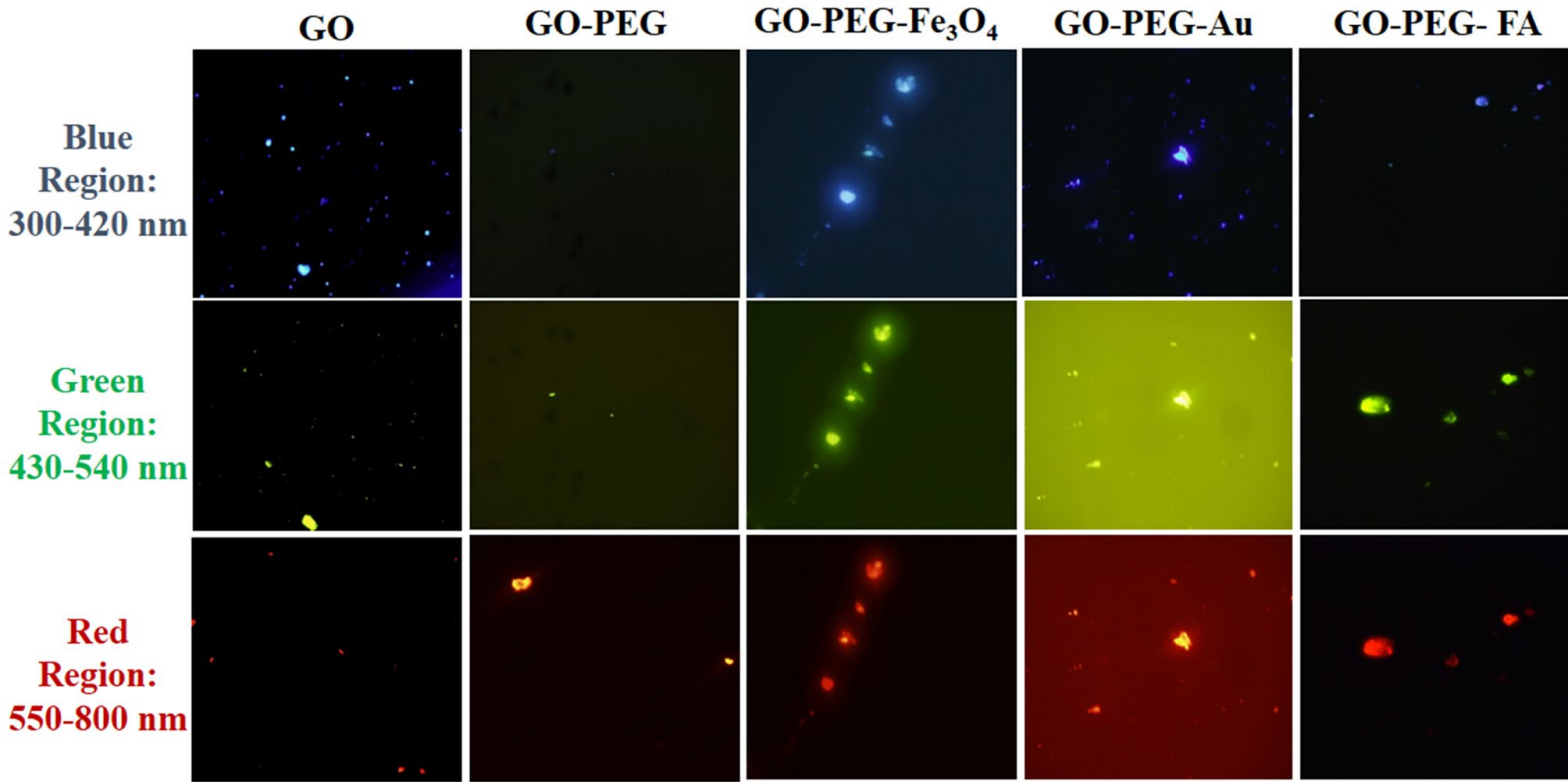
Fluorescence properties of GO, GO-PEG, GO-PEG-Fe_3_O_4_, GO-PEG-Au, and GO-PEG-FA observed by the fluorescence microscopy in three fluorescence regions: blue (300–420 nm), green (430–540 nm) and red (550–800 nm).

According to Figs. 9–12, the GO derivatives are potentially traceable fluorophores for imaging purposes in biological environments. Figure 13 shows the fluorescence images of modified GOs dissolved in PBS solution in three different fluorescence regions. After surface modification^73^, the photoluminescence property of GO differs, either quenched or accelerated, disregarding the applied excitation wavelength. PEG polymer quenches the GO emission in blue and green regions, while the red emission is still detectable. In fact, modified conjugates with metal nanoparticles are stronger at all three fluorescence regions, leading to the detectable images. The red shift occurred with folate makes this derivative an ideal sensor for bio-imaging purposes.

### Biocompatibility

**Figure 14:**
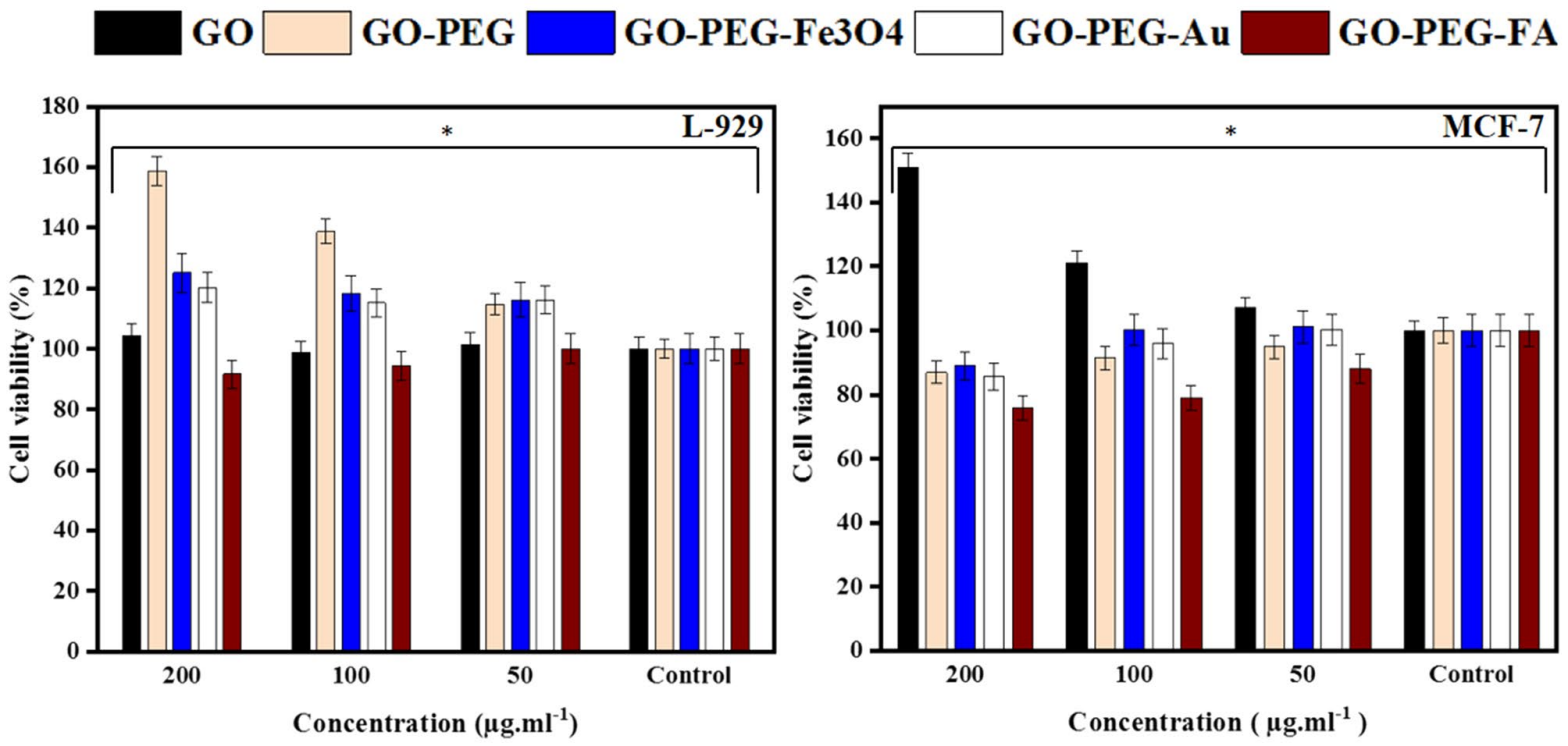
Viability assay of non-tumor cell lines (L-929) and tumor cell lines (MCF-7) treated with GO, GO-PEG, GO-PEG-Fe_3_O_4_, GO-PEG-Au, and GO-PEG-FA conjugates at different concentrations; Each test was repeated three time and p values were calculated using one-way ANOVA test (*p < 0.05).

MTT analysis was performed to evaluate the biocompatibility and cell toxicity of the prepared conjugates. GO, GO-PEG, GO-PEG-Fe_3_O_4_, GO-PEG-Au and GO-PEG-FA were tested against tumor (MCF-7) and non-tumor (L-929) cell lines at three different concentrations. From Fig. 14, both treated cells showed no appreciable viability loss after 48 h treatment, demonstrating the excellent biocompatibility of the synthesized conjugates.

## Conclusion

GO derivatives red shifted to the longer wavelengths, when the excitation occurred between 300 to 480 nm, representing the excitation-wavelength-dependent photoluminescence of GO. Such a behavior was the indicative of multiple fluorophore systems with aromatic and oxidation groups. The photoluminescence of modified GOs was mostly tunable at the excitation wavelength of 300 nm; the intrinsic fluorescence of both GO and PEG was not detected, when GO-PEG dissolved in water or DMEM cell media. This was due to the role of GO and PEG as the acceptor and donor, which quenches the individual fluorescence of each component. In contrast, at the physiological condition (pH = 7.4), the fluorescence of both GO and PEG was deserved. Fe_3_O_4_ and Au NPs, on the other hand, quenched the fluorescence property of PEGylated GO in acidic conditions. This characteristic preserves the detected fluorescence in cell media, deserving GO-PEG-Metal NPs as a potential switch-off biosensor in the tumor area. GO-PEG-FA however shows strong intensities at three regions, mostly green area, in all treated solutions.

The fluorescence of GO and PEG is totally quenched when GO-PEG and GO-PEG-metal NPs are excited at 350 nm wavelength (switch-off system). Whereas folate causes the blue shift in the emitted peaks.

When the excitement occurs at 430 nm, all four modified GO conjugates experienced interesting alterations. The observed red shift introduced GO-PEG ideal as the "switch-on biosensor". Metal NPs conjugates showed the multiple emission peaks, whereas folate quenched the predicated emissions at both blue and far red regions in GO-PEG-FA.

At the excitation of 480 nm, GO-PEG experienced a sharp peak and red shift in natural and acidic conditions, while metal NPs, in particular Au, as well as folate, accelerated the expected emission, representing GO-PEG-Au and GO-PEG-FA as the ideal theranostic agents.

GO and its derivatives in conclusion displayed an excited–state protonation of COOH groups in various pH conditions. The creation of localized *sp*^2^ clusters and structural defects during GO reduction through the modification were more likely to be responsible for the enhancement of green fluorescence. Besides, it was found that the fluorescence of GO and its derivatives conjugates were tunable between ultraviolet, visible and NIR with a robust intensity. These features suggested that the fluorescence aspect of GO, specifically at the excitation of 480 nm could be readily incorporated in a variety of biomedical imaging applications; Likewise, GO may act not only as a fluorophore, it can also operate as a quencher, introducing the new chances for the next generation biosensors with multiplex detections.
