# Author Correction: Graphene oxide and its derivatives as promising *In-vitro* bio-imaging platforms

**DOI:** 10.1038/s41598-021-88760-0

**Published:** 2021-04-21

**Authors:** Yasaman Esmaeili, Elham Bidram, Ali Zarrabi, Abbas Amini, Chun Cheng

**Affiliations:** 1 Faculty of Advanced Sciences and Technologies, University of Isfahan, Isfahan, Iran; 2 Biosensor Research Center, Department of Biomaterials, Nanotechnology, and Tissue Engineering, School of Advanced Technologies in Medicine, Isfahan University of Medical Sciences, Isfahan, Iran; 3 Nanotechnology Research and Application Center (SUNUM), Sabanci University, 34956 Tuzla, Istanbul Turkey; 4 Department of Mechanical Engineering, Australian College of Kuwait, 13015 Mishref, Safat Kuwait; 5 Department of Materials Science and Engineering, Southern University of Science and Technology, Shenzhen, China

Correction to: *Scientific Reports* https://doi.org/10.1038/s41598-020-75090-w, published online 22 October 2020

This Article contains an error in the horizontal axis labels of Figures 9, 10, 11 and 12 where “Wavenumber” should read “Wavelength”. The correct Figures 9, 10, 11 and 12 appear below as Figure 1, 2, 3 and 4.

**Figure 1:**
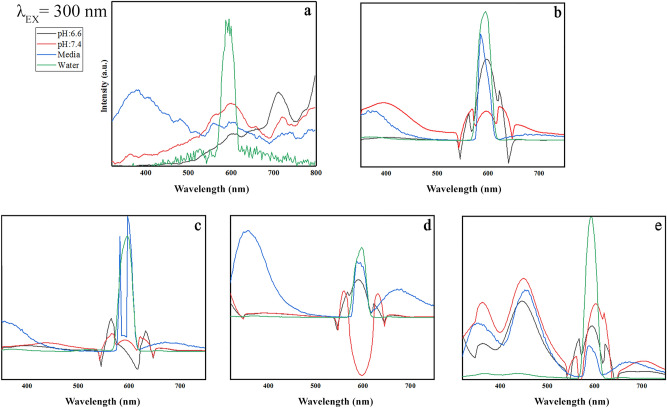
Emission patterns of (**a**) GO, (**b**) GO-PEG, (**c**) GO-PEG-Fe_3_O_4_, (**d**) GO-PEG-Au, and (**e**) GO-PEG-FA at the excitation wavelength of 300 nm in water, cell media (DMEM) and PBS, at two different pH values (pH 6.6 and 7.4).

**Figure 2:**
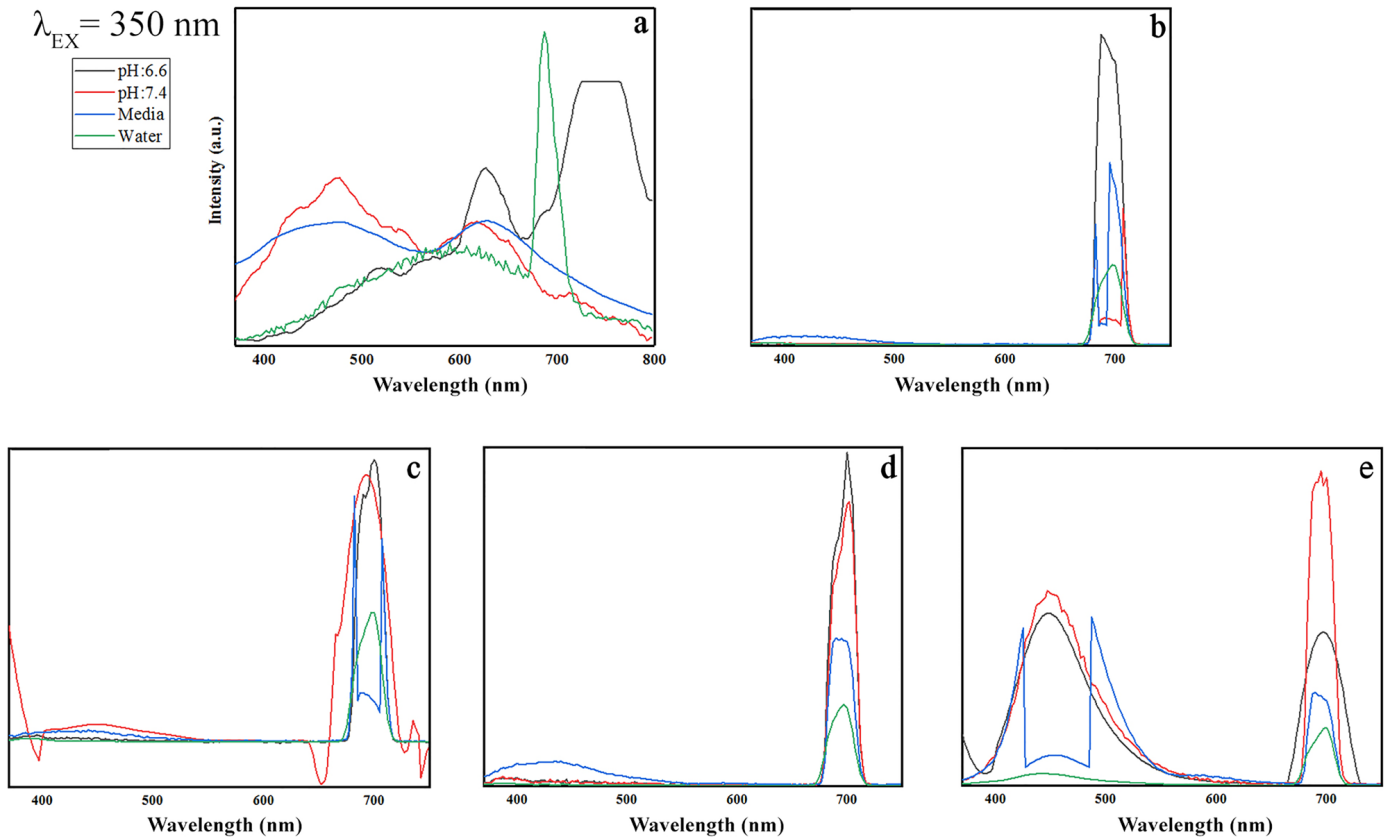
Emission patterns of (**a**) GO, (**b**) GO-PEG, (**c**) GO-PEG-Fe_3_O_4_, (**d**) GO-PEG-Au, and (**e**) GO-PEG-FA at the excitation wavelength of 350 nm in water, cell media (DMEM), and PBS at two different pH values (pH 6.6 and 7.4).

**Figure 3:**
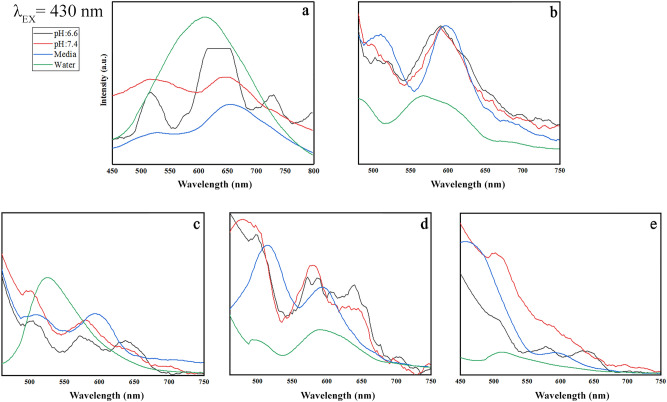
Emission patterns of (**a**) GO, (**b**) GO-PEG, **c**) GO-PEG-Fe_3_O_4_, (**d**) GO-PEG-Au, and (**e**) GO-PEG-FA at the excitation wavelength of 430 nm in water, cell media (DMEM), and PBS at two different pH values (pH 6.6 and 7.4).

**Figure 4:**
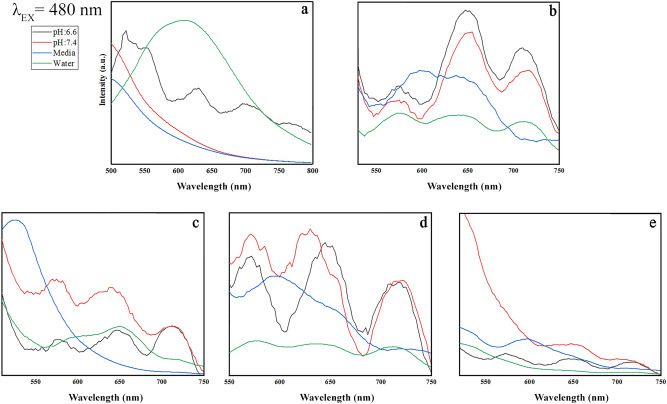
Emission pattern of (**a**) GO, (**b**) GO-PEG, (**c**) GO-PEG-Fe_3_O_4_, (**d**) GO-PEG-Au, and (**e**) GO-PEG-FA at the excitation wavelength of 480 nm in water, cell media (DMEM), PBS at two different pH values (pH 6.6 and 7.4).

